# Combining transcranial magnetic stimulation with training to improve social cognition impairment in schizophrenia: a pilot randomized controlled trial

**DOI:** 10.3389/fpsyg.2024.1308971

**Published:** 2024-02-20

**Authors:** Alessandra Vergallito, Bianca Gramano, Kevin La Monica, Luigi Giuliani, Davide Palumbo, Camilla Gesi, Sara Torriero

**Affiliations:** ^1^Department of Psychology and Neuromi, University of Milano-Bicocca, Milan, Italy; ^2^Department of Mental Health and Addictions, ASST Fatebenefratelli-Sacco, Milan, Italy; ^3^Department of Psychiatry, Università degli Studi della Campania "Luigi Vanvitelli", Naples, Italy

**Keywords:** schizophrenia, social cognition, TMS, iTBS, DLPFC

## Abstract

Schizophrenia is a severe, chronic mental disorder that profoundly impacts patients’ everyday lives. The illness’s core features include positive and negative symptoms and cognitive impairments. In particular, deficits in the social cognition domain showed a tighter connection to patients’ everyday functioning than the other symptoms. Social remediation interventions have been developed, providing heterogeneous results considering the possibility of generalizing the acquired improvements in patients’ daily activities. In this pilot randomized controlled trial, we investigated the feasibility of combining fifteen daily cognitive and social training sessions with non-invasive brain stimulation to boost the effectiveness of the two interventions. We delivered intermittent theta burst stimulation (iTBS) over the left dorsolateral prefrontal cortex (DLPFC). Twenty-one patients were randomized into four groups, varying for the assigned stimulation condition (real vs. sham iTBS) and the type of cognitive intervention (training vs. no training). Clinical symptoms and social cognition tests were administered at five time points, i.e., before and after the treatment, and at three follow-ups at one, three, and six months after the treatments’ end. Preliminary data show a trend in improving the competence in managing emotion in participants performing the training. Conversely, no differences were found in pre and post-treatment scores for emotion recognition, theory of mind, and attribution of intentions scores. The iTBS intervention did not induce additional effects on individuals’ performance. The methodological approach’s novelty and limitations of the present study are discussed.

## Background

Schizophrenia is a severe, disabling, and chronic mental disorder that affects approximately 24 million people worldwide (Institute of Health Metrics and Evaluation, 2019) and seriously impairs individuals’ functioning and quality of life. Patients often live an isolated and marginalized existence, with limited social contacts other than close relatives and without acquiring proper education, accommodation, income, and social or working skills (Chan and Yu, 2004; Solanki et al., 2008). Over the past two decades, cognitive impairment has become central in research and clinical interest due to its impact on patients’ everyday lives and activities (Sharma and Antonova, 2003; Fioravanti et al., 2012; Verma et al., 2012; Schaefer et al., 2013; Fervaha et al., 2014; Shmukler et al., 2015; Green et al., 2015a; Carbon and Correll, 2014). Among cognitive deficits, social cognition impairment showed a tighter association with patients’ troubles in everyday functioning than positive and negative symptoms (Brekke et al., 2005; Schmidt et al., 2011; Green et al., 2015b; Halverson et al., 2019). Since pharmacotherapy showed limited effects in alleviating cognitive impairment (Kucharska-Pietura and Mortimer, 2013; Na et al., 2015; McCleery and Nuechterlein, 2019), researchers focused on developing new interventions to improve patients’ social abilities.

Social cognition is a complex function that can be defined as the set of psychological processes involved in understanding social situations, building a narrative coherence concerning internal and external experiences, and producing appropriate behaviors in interpersonal situations (Harvey and Penn, 2010; Hasson-Ohayon et al., 2015; Green et al., 2015a). Research on social cognition in schizophrenia typically focused on four accepted domains: (i) *Emotion recognition*, namely inferring emotions – typically from facial expressions; (ii) *Theory of Mind*, namely the ability to understand other people’s mental states; (iii) *Social Perception*, i.e., identifying social contexts, roles and rules from non-verbal social cues such as voice intonation and body language; (iv) *Attributional bias*, or the ability to infer the causes of situations or behaviors [see for a recent review (Green et al., 2019)]. Previous studies suggested that schizophrenic patients show moderate to severe impairments in these domains compared to demographically matched healthy individuals (Kohler et al., 2010; Savla et al., 2013), although the first two areas (emotion recognition and theory of mind) have been more widely investigated than the others (Kurtz et al., 2016; Green et al., 2019). Social cognition impairments have been reported in individuals at risk for developing psychosis (Shakeel et al., 2019) and first-degree patients’ relatives (Lavoie et al., 2013; Martin et al., 2020). Deficits are present at the different stages of the disease (McCleery et al., 2016), from the prodromal phase (Green et al., 2012; Lee et al., 2015) and first-episode (Daros et al., 2014; Healey et al., 2016; Kuharic et al., 2019), they worsen with multiple psychotic episodes (Kuharic et al., 2019) and persist even when other symptoms improve (Green, 2016).

To improve social cognition impairment, researchers developed several psychosocial remediation interventions, with a specific interest in the long-term maintenance of changes and the possibility of transferring the improvements to real-life functioning (Wykes and Spaulding, 2011; Bowie et al., 2020; Mucci et al., 2021). Psychosocial interventions fall into three main categories: (i) targeted programs, which focus on a specific domain of social cognition; (ii) comprehensive programs, which try to target all the social cognition components impaired in schizophrenia; (iii) integrated programs, that combine training on nonsocial and social cognitive abilities [for recent reviews, see (Kimoto et al., 2019; Nijman et al., 2020)]. Meta-analyses on social cognitive interventions suggested that the treatments improve emotion recognition and theory of mind domains; however, the possibility of generalizing such effects in patients’ everyday lives produced mixed results (Kurtz and Richardson, 2012; Nijman et al., 2020).

Over the past years, non-invasive brain stimulation (NiBS) – particularly transcranial magnetic stimulation (rTMS) and transcranial direct current stimulation (tDCS) – received large attention in the treatment of psychiatric disorders (Hyde et al., 2022), among which schizophrenia. The rationale for applying NiBS in mental disorders is the possibility of modifying the altered neural plasticity by rebalancing pathological activity and maladaptive functional connectivity within brain regions (Nitsche et al., 2012b; Kronberg et al., 2017; Ziemann, 2017). Indeed, the two techniques act by modulating neuronal activity by depolarizing or hyperpolarizing specific cerebral areas, via trains of magnetic pulses in the case of repetitive TMS (rTMS) (Hallett, 2000; Fregni and Pascual-Leone, 2007) or weak electrical currents in tDCS (Nitsche et al., 2008), with biochemical processes that outlast the time of stimulation (Reis et al., 2008; Cirillo et al., 2017).

International expert-panel guidelines based on meta-analyses of available studies (Lefaucheur et al., 2020) suggested Level C (possibly effective) recommendations for the application of low-frequency repetitive transcranial magnetic stimulation (rTMS) over the left temporoparietal cortex to treat auditory hallucinations and of high-frequency rTMS over the left dorsolateral prefrontal cortex (DLPFC) to improve negative symptoms. Previous research using NiBS to target patients’ nonsocial cognitive impairment produced heterogeneous results (Iimori et al., 2019; Jiang et al., 2019; Sloan et al., 2021; Goh et al., 2022), suggesting that effects – when traceable – are typically confined to specific functions such as episodic-immediate memory (Guan et al., 2020; Xiu et al., 2020; Wen et al., 2021).

Concerning the application of NiBS to modulate social cognition, previous studies investigated such possibility in healthy and clinical populations [see for reviews (Marini et al., 2018; Nejati et al., 2022; Yamada et al., 2022)]. Stimulation protocols primarily focused on prefrontal regions – especially the dorsolateral prefrontal cortex (DLPFC) and medial prefrontal cortex (MPFC) – and the temporoparietal junction (TPJ) (Schuwerk et al., 2014). Indeed, processes involved in social cognition involve an extensive network of regions encompassing prefrontal, temporoparietal, and limbic structures [for reviews see (Green et al., 2015a; Arioli et al., 2018; Kimoto et al., 2019)]. In particular, the lateral prefrontal cortex seems to be involved in the cognitive (vs. affective) component of social cognition, including cognitive control of emotional information triggered by facial expressions (Zaki et al., 2010; Vanderhasselt et al., 2013a,b), mentalizing abilities (Abu-Akel and Shamay-Tsoory, 2011), or assuming others’ perspective (Conson et al., 2015).

In previous studies anodal tDCS over the left DLPFC improved facial emotion recognition in healthy participants (Nitsche et al., 2012a) and a similar effect was found in depressed patients after 20 high-frequency rTMS sessions (Tong et al., 2021). Considering schizophrenic patients, only a few research previously applied NiBS to target social cognition. In a first study, Rassovsky et al. (2014) applied single pulses of TMS to investigate if patients’ impairment in facial recognition might be due to low-level visual processing. The authors manipulated pictures depicting facial expressions by modifying the image spatial frequencies, and TMS pulses were applied over the primary visual cortex before or after the stimuli presentation in schizophrenic patients vs. healthy controls. Participants were asked to recognize the correct emotion among pictures depicting happy, sad, afraid, and angry expressions. Findings confirmed patients’ general impairment in recognizing facial expressions compared to healthy controls. However, no differences were found between controls and patients when considering the manipulation of the pictures, suggesting that patients are not impaired in low-level processing but may be compromised at a later integrative stage.

The subsequent studies, differently from the one by Rassovsky and colleagues, applied NiBS with an intervention purpose, namely to modulate performance in social cognition tasks. In line with the neural networks previously described, the stimulation protocols targeted the DLPFC. Wolwer and colleagues (Wölwer et al., 2014) randomized 32 patients with chronic schizophrenia in two groups receiving 10 sessions of real or sham 10-Hz rTMS delivered over the left DLPFC. The protocol lasted two weeks, and a facial affect recognition task was performed at baseline and immediately after the treatment. The authors reported that all participants improved at the post-treatment evaluation, but the improvement was larger in the group assigned to the real stimulation condition. In another study, Rassovsky et al. (2015) randomized 36 patients into three tDCS conditions, in which participants received a single session of 1 mA anodal, cathodal, or sham tDCS. Stimulation was delivered for 20 min (30 s for the sham condition) over the bilateral DLPFC, with a reference electrode applied over the upper right arm. The researchers administered tasks evaluating different domains of social cognition, namely facial emotion identification, theory of mind, social perception, and emotional intelligence. Findings highlighted that participants receiving anodal stimulation improved after tDCS only in the emotion identification task. No differences were traceable for cathodal and sham stimulation or tasks evaluating the other components of social cognition. Finally, in a recent study Jin et al. (2023) applied an accelerated intermittent theta burst stimulation (iTBS) protocol over the left DLPFC. Participants received three daily stimulation sessions (1,800 pulses), with an interval of 15 min, for four weeks. The researchers administered a facial emotion recognition task and a theory of mind verbal task and evaluated clinical symptoms before and after the treatment. Results highlighted that the group receiving real iTBS improved after treatment in the social cognitive domains and negative symptoms compared to the sham iTBS group.

Taken together, the studies using NiBS to improve nonsocial and social cognition functions suggested the feasibility of this approach. However, two main limitations emerged from the literature review. First, previous works applied the stimulation at resting state without time-locking stimulation with behavioral or cognitive tasks engaging the same neural network. Over the past years, however, experimental neuroscience provided compelling evidence on the state-dependency of NiBS effects (Silvanto et al., 2008; Pisoni et al., 2018; Borgomaneri et al., 2020; Sack et al., 2023; Vergallito et al., 2023b); therefore, the possibility of combining psychological, cognitive, or behavioral interventions with time-locked brain stimulation seems to be a promising approach to boost through NiBS the spontaneous neuroplastic changes induced by the training *per se* and rebalancing pathological brain activity and connectivity patterns (Sathappan et al., 2019; Gainsford et al., 2020; Dedoncker et al., 2021; Vergallito et al., 2021; Tatti et al., 2022). A second limitation concerns the lack of follow-up evaluations. Indeed, only a few studies investigated rTMS effects at longer intervals than the post-intervention time point (Li et al., 2016; Francis et al., 2019; Xiu et al., 2020; Voineskos et al., 2021; Du et al., 2022). Interestingly, some of these studies provided preliminary evidence concerning stimulation-delayed effects that were traceable at follow-ups but not immediately after the end of the stimulation protocol (Freitas et al., 2009; Li et al., 2016; Xiu et al., 2020; Du et al., 2022).

### Aims of the present study

The present study is a pilot randomized controlled trial, in which we used iTBS as a primer aiming to augment the effect of individualized training in nonsocial and social cognition domains in schizophrenic patients. Participants were randomized in four groups, in which they received real vs. sham iTBS combined (or not) with the training.

The choice of stimulating the left DLPFC is based on the previously reviewed literature suggesting improvements in emotion recognition (Nitsche et al., 2012a; Wölwer et al., 2014; Tong et al., 2021; Jin et al., 2023), although the mechanisms underlying this effect remain largely unknown (Jin et al., 2023). It is possible that iTBS (or ‘excitatory’ NiBS protocols) increases the activity and metabolism of the left DLPFC, which are reduced in schizophrenic patients [see for a review (Smucny et al., 2022)], and improves its connectivity with other regions involved in social cognition, among which the frontoparietal network (Schurz et al., 2020), which has been shown to be impaired in this population (Deserno et al., 2012; Fryer et al., 2015; Ćurčić-Blake et al., 2022). Indeed, while brain stimulation has been initially considered as primarily affecting the stimulated region, it is now clear that it can also influence remote brain areas interconnected with the stimulation site (Ruff et al., 2009; Romero Lauro et al., 2014, 2016; Pisoni et al., 2018; Beynel et al., 2020).

In the present study iTBS was selected since it requires a shorter time of stimulation compared to high frequency rTMS protocols (3 min vs. 20–40 min) (Suppa et al., 2016) and it seems to induce long lasting effects, up to 60 min (Wischnewski and Schutter, 2015), that could cover the time required for our training.

Clinical symptoms, functional scales, and nonsocial and social cognition tasks were assessed at baseline and after the treatment, plus in three follow-ups at one, three, and six months after the treatment ended.

In the present work, we present the methodological approach and preliminary data on social cognition measures, whereas iTBS’s impact on clinical symptoms and nonsocial cognitive functions is presented in another work (Vergallito et al., 2023a).

## Materials and methods

### Participants

**Figure 1:**
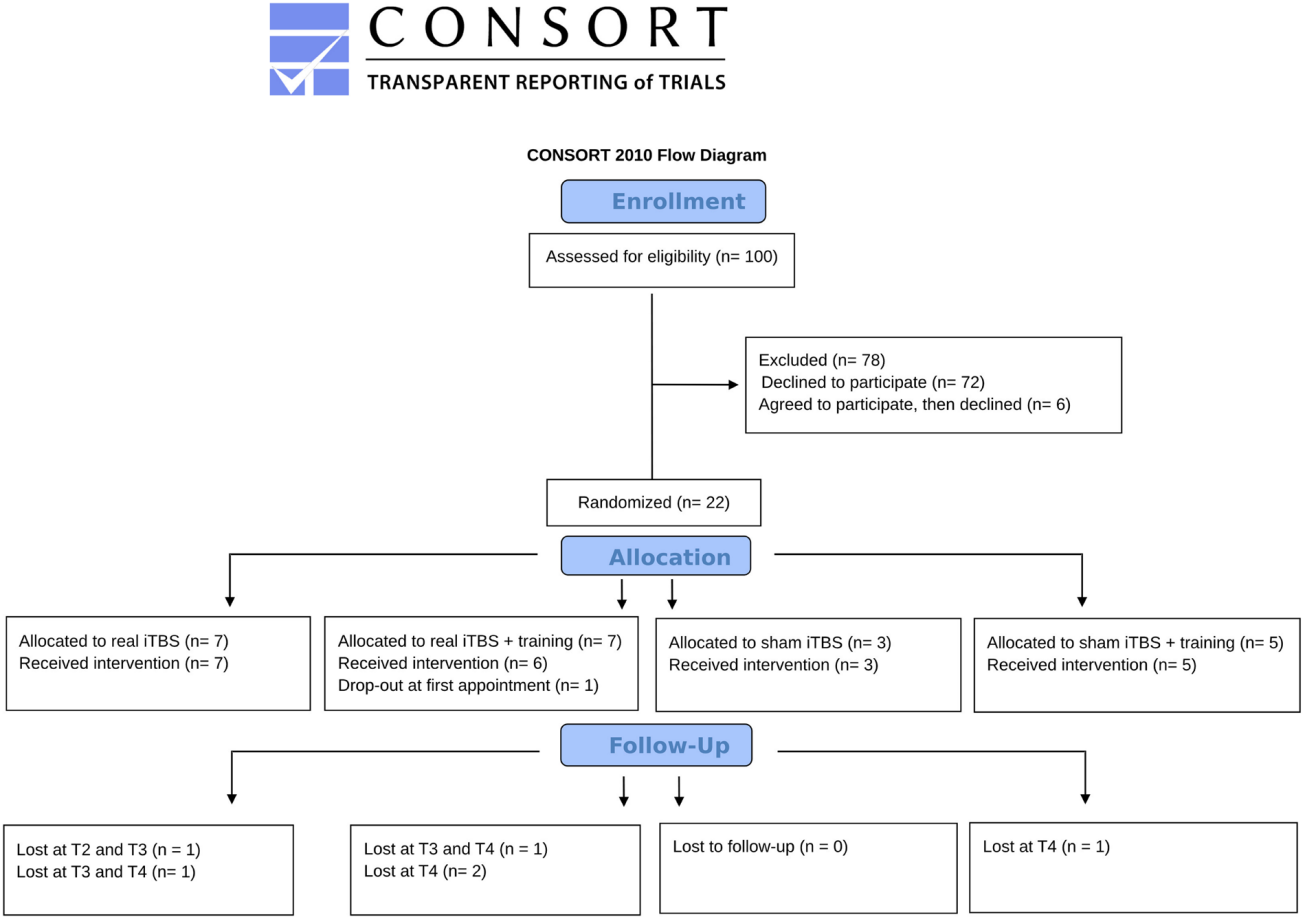
The CONSORT flowchart (Schulz et al., 2010) includes the number of participants in each group and phase.

One hundred eligible patients from the outpatient facilities of the ASST Fatebenefratelli-Sacco and Fondazione IRCCS San Gerardo dei Tintori were invited to participate in the project between July 2020 and July 2023. In the original proposal, we foresaw collecting at least 40 patients (10 per group), but it was impossible due to the COVID-19 pandemic waves and the high rate of patients declining to participate (see Figure 1).

Twenty-one participants (16 males, mean age = 35.2, SD = ± 10.8, mean illness duration years = 10, SD = 8.8) completed the three-week intervention. Inclusion criteria were: (i) age between 18 and 60; (ii) a diagnosis falling into the schizophrenia spectrum according to the Diagnostic and Statistical Manual of Mental Disorders, fifth edition (DSM-5) criteria; (iii) stable on medication for at least 3 months. Exclusion criteria were: (i) contraindications to TMS (neurological disorders, pregnancy, pacemaker); (ii) substance dependency within the previous six months; (iii) inability to provide informed consent. All participants gave written informed consent before the study procedure. The local ethics committee approved the study (2018/ST/081), and participants were treated following the Declaration of Helsinki.

Table 1 summarizes participants’ demographic and baseline clinical features.

**Table 1:** Summarizes the baseline demographic and clinical characteristics of included participants.

Variable	iTBS (*N* = 7)	iTBS + Training (*N* = 6)	Sham (*N* = 3)	Sham + Training (*N* = 5)
*Demographic*				
Gender (F/M)	2/5	1/5	2/1	0/5
Age	38.3 ± 11.2	36.3 ± 13.8	32.3 ± 8.3	31.2 ± 8.9
Education	12.3 ± 1.9	12.2 ± 2.0	12.3 ± 1.2	11.4 ± 2.1
Illness duration (years)	11.1 ± 10.7	13.3 ± 11.0	4.7 ± 0.6	7.6 ± 4.3
*Diagnosis*				
Schizophrenia	5	4	2	3
Schizoaffective disorder	1	1	1	1
Psychotic disorder NOS	1	1	–	1
*Clinical measures*				
PANSS	62.6 ± 22.7	71.0 ± 24.8	70.3 ± 6.5	53.6 ± 8.0
BNSS	25 ± 19.7	30 ± 22.7	24.7 ± 11.9	18.6 ± 8.0
CDSS	5.3 ± 5.0	6.2 ± 3.4	8.0 ± 5.3	4.4 ± 1.1
CGI	3.6 ± 1.5	3.8 ± 1.5	3.7 ± 0.6	2.8 ± 0.8
SLOF	185 ± 24.5	166.5 ± 36.1	165.3 ± 27.2	193.2 ± 15.7
WHOQOL “quality” – item G1	3.16 ± 1.5	3.8 ± 1.0	3.7 ± 0.6	3.8 ± 0.4

Notes: BNSS = Brief Negative Symptom Scale, CDSS = Calgary Depression Scale for Schizophrenia, iTBS = intermittent theta burst stimulation, CGI = Clinical Global Impression, NOS = Not Otherwise Specified, PANSS = Positive and Negative Syndrome Scale, SLOF = Specific Level of Functioning, WHOQOL = World Health Organization Quality of Life Assessment.

The pharmacological treatments were in adequate and stable doses (at least three months before protocol inclusion) and were maintained throughout the study. Therapies included antipsychotics (*n* = 19), antidepressants (*n* = 19), mood stabilizers (*n* = 6), anti-epileptics (*n* = 7), and benzodiazepines (*n* = 7).

### Standardized tasks investigating abilities of social cognition

- The Mayer-Salovey-Caruso Emotional Intelligence Test – Managing Emotions (MSCEIT-ME) (Mayer, 2002) is a social cognition subtest included in the MATRICS Consensus Cognitive Battery (MCCB) (Kern et al., 2008; Nuechterlein et al., 2008), which measures emotional intelligence and specifically the ability to manage and regulate own and others emotions. The test branch consists of eight written stories, each presenting three different solutions, whose effectiveness had to be evaluated by participants. The T-score of MSCEIT-ME from MCCB was used for analyses, where higher scores indicate higher emotional intelligence.
- The Facial Emotion Identification Task (FEIT) (Kerr and Neale, 1993) was administered to evaluate the emotion recognition domain. The FEIT includes 58 pictures of faces depicting one out of six different emotions (happiness, sadness, anger, surprise, disgust, fear) or a neutral expression. Participants see one picture at a time for 15 s and make a forced choice to select the proper emotion. The test score comprises the total number of trials correctly identified by participants.
- The Awareness of Social Inference Test (TASIT) (McDonald et al., 2003) investigates the Theory of Mind (ToM) abilities through videotaped vignettes representing everyday social interactions. In the present experiment, we administered only the third section of the TASIT, namely the Social Inference-Enriched test (SI-E) (Ludwig et al., 2017). TASIT SI-E includes 16 vignettes in which participants receive information about events occurring before or after the represented dialog that help contextualize characters’ interaction. Specifically, the test investigates the comprehension of lie and sarcasm. Higher scores on this task indicate higher theory of mind skills.
- The Ambiguous Intentions Hostility Questionnaire (AIHQ) (Combs et al., 2007) is a self-administered questionnaire comprising 15 written stories describing negative social actions performed by others. The vignettes differ according to the clarity of individuals’ reasons and, specifically, whether the events occur accidentally, intentionally, or ambiguously (five for each possible scenario). After each scene, participants have to complete three anchored questions concerning the agent’s intention and blameworthiness and participants’ anger. In response to each vignette, the self-report total score (blame score) was totaled. Higher scores in this task indicate the tendency to interpret as hostile others’ intentions. A subscale including only mean scores of ambiguous situations (AIHQ_AMB) was computed.

### Standardized clinical scales

- The Positive and Negative Syndrome Scale (PANSS) (Kay et al., 1987) assesses illness severity. PANSS includes a 30-item scale investigating three major dimensions, namely *positive symptoms* (7 items examining delusions, hallucinations, conceptual disorganization, grandiosity, suspiciousness/persecution, hostility), *negative symptoms* (7 items measuring blunted affect, emotional withdrawal, poor rapport, passive/apathetic social withdrawal, difficulty in abstract thinking, lack of spontaneity and flow of conversation, stereotyped thinking) and *general psychopathology* (16 items investigating somatic concern, anxiety, guilt feelings, tension, mannerisms and posturing, depression, motor retardation, uncooperativeness, unusual thought content, disorientation, poor attention, lack of judgment and insight, disturbance of volition, poor impulse control, preoccupation, and active social avoidance). Items are on a 7-point Likert scale where 1 indicates the absence of symptoms, and 7 indicates extremely severe symptoms.
- The Italian version of the Brief Negative Symptom Scale (BNSS) (Kirkpatrick et al., 2011; Mucci et al., 2015) was used to assess negative symptoms. The scale measures the five domains indicated by the NIMH Consensus Development Conference (Kirkpatrick et al., 2006) as essential parts of the negative dimension, namely affective flattening, alogia, anhedonia, avolition, and asociality, allowing the assessment of the two principal dimensions of motivation/pleasure and emotional expressivity. Higher scores in this questionnaire indicate higher negative symptoms.
- The Calgary Depression Scale for Schizophrenia (CDSS) (Addington et al., 1993) was administered to assess the level of depression. The scale has been developed to measure depression in schizophrenic patients and includes nine clinician-rated items. Higher scores on this scale indicate higher depressive symptoms.
- The Clinical Global Impression (CGI) (Guy, 1976), completed by the clinician, was used to assess the current severity of illness on a 7-point Likert scale, where higher rates represent high-severity illness.
- The Specific Level of Functioning (SLOF) (Schneider and Struening, 1983; Mucci et al., 2014) was adopted to measure the patients’ functioning. Informed caregivers or care workers completed the scale based on observing the patient’s behavior and functioning in different domains: self-care, social functioning and community abilities.
- The World Health Organization Quality of Life Assessment (WHOQOL-BREF) (Group, 1998) was administered to measure patients’ perceived quality of life. This self-administered questionnaire includes 26 items that measure satisfaction in 4 life domains: physical health, psychological, social relationships, and environmental. Higher scores indicate a greater perceived quality of life.

### TMS parameters

Stimulation was administered using a Magstim Rapid2 magnetic biphasic stimulator connected to a 70-mm diameter figure-of-eight coil (Magstim Company, Whitland, United Kingdom).

In each stimulation session, TMS was delivered following the iTBS pattern described by Huang et al. (2005), a protocol that rapidly induces a long-term potentiation process similar to synaptic plasticity (Huang et al., 2005; Rossini and Rossi, 2007), consisting of 2 s trains of TBS (3 TMS pulses delivered at 50 Hz repeated every 200 ms) delivered every 10 s (2 s stimulation and 8 s of intertrial interval).

Participants received 20 iTBS trains, for a total of 600 pulses per session (190 s). Stimulation intensity (M = 41.2, SD = 5.7) was set at 100% of active motor threshold (AMT), which is defined as the lowest intensity of the stimulator output inducing motor evoked potentials (MEPs) with an amplitude of at least 100 μV in the contralateral first dorsal interosseous muscle, with a 50% of probability during an isometric contraction of ~10–20% of the maximum voluntary contraction of the muscle (Rossini et al., 1994). During the iTBS, the coil was positioned over the left DLPFC (F3 according to the 10–20 EEG system) and constantly monitored using the Softaxic neuronavigation system (Version 3) (EMS, Bologna, Italy) combined with a Polaris Vicra infrared camera (NDI, Waterloo, Canada), to ensure consistency of coil placement across days. Real iTBS was applied using a figure-of-eight coil held tangentially to the scalp with the handle pointing posteriorly, whereas sham stimulation was applied placing the coil at 90° from the scalp.

Participants received 15 daily sessions of real or sham stimulation (5 consecutive working days for 3 weeks), followed or not by nonsocial and social cognition training.

### Social cognition training

The training targeting social cognition abilities always followed the cognitive one. The cognitive training was performed individually with the experimenter (A.V.) by using the software COGPACK (version 9.3, Marker Software, Ladenburg, Germany), described in detail in another work (Vergallito et al., 2023a). Materials from the Social Cognition Individualized Activities Lab (SoCIAL) (Palumbo et al., 2017, 2022) were used to train social cognition abilities. The Emotion recognition module was administered in the first seven sessions. The module aimed to improve individuals’ abilities in discriminating between different emotional states by employing static pictures depicting facial emotional expressions and videos representing dynamic situations. The pictures presented basic emotions (anger, fear, happiness, surprise, sadness, disgust) at different intensities so that some faces were more ambiguous than others. The aim of showing pictures was to train (or teach) participants to recognize facial details to improve their ability to detect emotions. Moreover, videos representing emotions allowed patients to recognize and integrate micro-expressions, prosody, and gestures in a dynamic situation, with actors representing different emotions and to different degrees along the videos.

The ToM Module was administered in the following 8 sessions. In this module, participants saw 16 videos in which they learned to identify social and contextual cues to understand the mental state of actors. Specifically, videos were coupled with identical or similar scripts. One version of each video had a literal meaning, while the other included social cues (e.g., gestures, voice intonation) conveying a nonliteral meaning, for example, sarcasm, disappointment, and so on. For each video, previously prepared questions were used to prompt the discussion.

### Procedure

Once they accepted to participate in the study, patients were submitted to a baseline evaluation, including neuropsychological and clinical batteries. The evaluation was performed by a trained psychotherapist with neuropsychological expertise (S.T.). It was scheduled in two sessions to avoid the fatigability of patients: in the first session, clinical scales and social cognition tests were administered, while the second session was dedicated to the cognitive assessment through the MCCB. The week after the baseline evaluation, participants started a three-week treatment. They were randomly assigned to 4 different groups, receiving real or sham iTBS combined or not with the cognitive and social training (groups: iTBS + training; sham iTBS + training; iTBS - no training; sham iTBS - no training). Randomization was done by using the RAND function in Excel (numbers 1–4 each representing an experimental condition) on the foreseen sample (n = 40). Since the sample was not completed, the groups’ sizes show some differences, with one group (sham iTBS – no training) with a smaller sample size than the others.

The study was single-blind: participants were inevitably aware whether they were allocated to the group receiving the training or not, but they did not know whether they were assigned to the group receiving real or sham stimulation. The experimenters involved in patients’ evaluations and training were not blind. Participants were not informed about the stimulation condition, which was directly communicated to the experimenter administering the iTBS protocol. The included patients did not experience TMS before their participation in the study.

All scales completed at baseline (T0) were then repeated at the end of the treatment (T1) and at three follow-ups at one, three, and six months after treatment’s end (T2, T3, T4). When available (i.e., FEIT and TASIT), social cognition tasks were administered alternating two parallel forms across time intervals.

### Primary and secondary endpoints

The primary aim of our study was to investigate whether a multimodal approach combining iTBS and training (iTBS + training) could improve social cognition abilities, compared to the other experimental conditions in which participants received only iTBS (iTBS – no training), training (sham iTBS + training) or no intervention (sham iTBS – no training). The primary endpoints, therefore, included pre-post scores at MSCEIT-ME, FEIT, TASIT, and AIHQ tests.

Our secondary aim was to explore the durability of stimulation effects, investigating possible delayed effects at one, three, and six months after the end of the treatment. Moreover, baseline correlations between clinical and functional measures and social cognition abilities were explored. To avoid redundancy, we will present primary and secondary endpoints together for the same outcome measures, and correlation analyses will be presented in a separate section.

### Statistical analysis

Data were analyzed in the statistical programming environment R (R Core Team, 2023). The datasets and the script generated for the analyses are publicly available at https://osf.io/d5aeb/. Scores from the different social cognition tasks were analyzed using linear mixed-effects models (Baayen et al., 2008; Muth et al., 2016), fitted using the LMER function of the lme4 R package (Bates et al., 2015).

Considering the primary endpoint, the predictors’ time (2 levels: before vs. after treatment), group (2 levels: iTBS vs. sham iTBS), training (2 levels: training vs. no training), and their interaction were entered in the full model as fixed factors. Considering analyses including follow-up scores, the only difference concerned the variable time (5 levels: before vs. after treatment, one, three, and six months after treatment end). The by-subject random intercept was included to account for individuals’ variability. The inclusion of predictors in the final models has been tested with a series of likelihood ratio tests (LRT) by progressively removing parameters that did not significantly improve the overall model goodness of fit (Gelman and Hill, 2006). Details on the model selection are available in the Supplementary materials (Section A). *Post-hoc* analyses were performed for significant interactions using the testInteractions function of the phia package (De Rosario-Martinez et al., 2015). The package ggplot2 (Wickham, 2011) was used for data visualization.

Correlations were run to investigate specific relationships among clinical symptoms, functioning scales, and social cognition variables at baseline. Pearson correlation coefficients and two-tailed probabilities applying Bonferroni correction were computed. The correlation matrix was plotted using the corrplot package (Wei et al., 2017).

## Results

### Regression analysis

One participant assigned to the group iTBS + training performed only the MSCEIT-ME task instead of the complete social cognition evaluation due to his high fatigability. The same occurred for two other participants at follow-ups (T4 for a participant assigned to the iTBS – no training group; T3 for a participant assigned to the sham iTBS + training group). Participants that dropped-out at follow-ups are reported in Figure 1.

**Figure 2:**
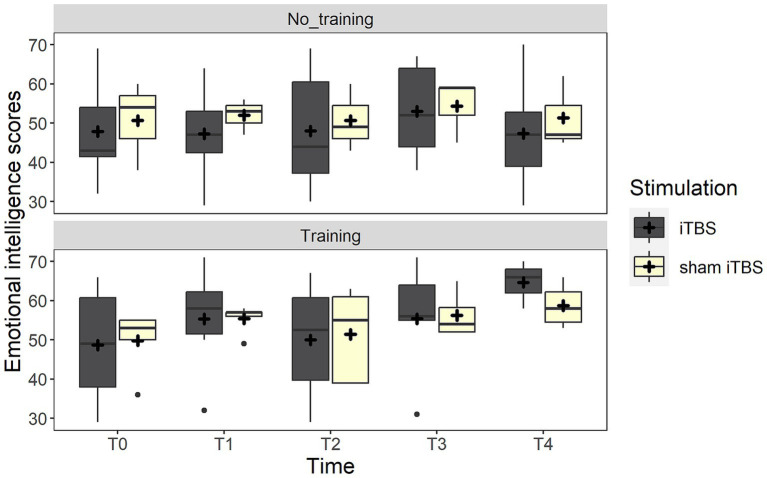
The figure depicts the emotional intelligence scores (MSCEIT) at the different time points. The boxplots represent the comparison between real iTBS (dark gray boxes) and sham iTBS (light yellow boxes) separately for the no training and training conditions.

Considering the MSCEIT-ME score, the best-fitting model included a trend in the interaction between training and time (χ2_(1)_ = 3.2, *p* = 0.072). *Post-hoc* analyses highlighted that only participants assigned to the group receiving the training improved after treatment (*p* = 0.009), while no differences were found in the no-training group (*p* = 1). Crucially, the two groups did not differ at baseline (*p* = 0.919). The interaction, however, disappeared when adding the three follow-up measures (all ps >0.170) (see Figure 2).

**Figure 3:**
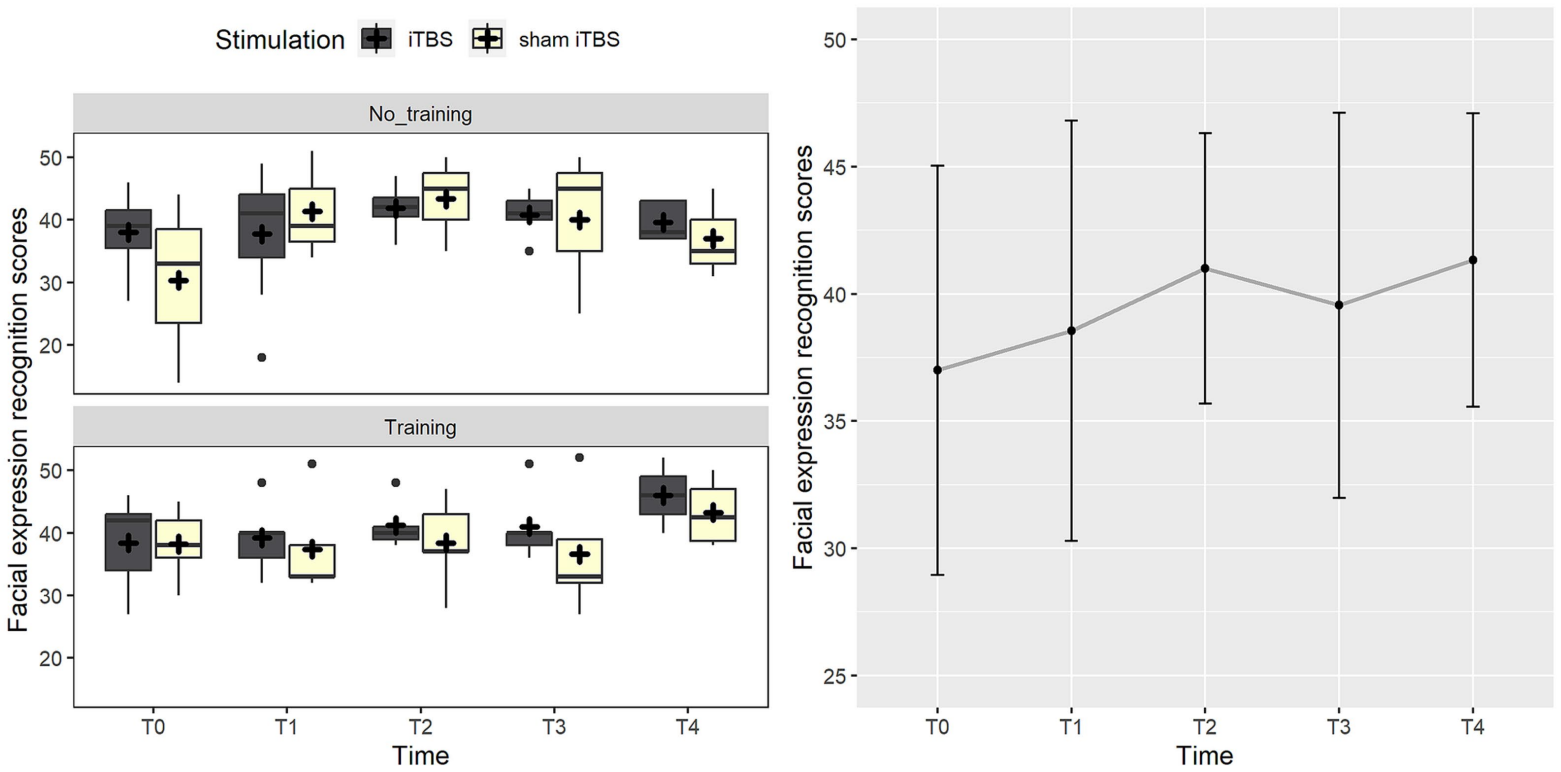
The figure represents the effects on the facial expression recognition score (FEIT). On the left panel, data trend for the real iTBS (dark gray boxes) and sham iTBS (light yellow boxes) at the different time points is reported. On the right panel, the graph shows performance at FEIT at the different time points.

Considering the FEIT scores, the best-fitting model included the three-way interaction between stimulation, training, and group (*χ*^2^_(1)_ = 3.8, *p* = 0.050). *Post-hoc* analysis highlighted that the group receiving sham stimulation with no training improved at the post-treatment evaluation (*p* = 0.007). Such an effect may be probably due to the worst performance of a participant at T0 in this group, as highlighted by Supplementary Figure S1 (Supplementary materials), which represents individual trends. The other comparisons were not significant (*p* > 0.193). Such an effect was not confirmed when follow-up measurements were added to the analysis. In this case, a trend toward significance suggested the inclusion of time (*χ*^2^_(4)_ = 8.4, *p* = 0.077), where the evaluation at T2 showed a trend toward improvement compared to the baseline (*p* = 0.071), probably as an effect of the task repetition since the assessment was repeated in a shorter time (every month) up to T2 and then with longer time intervals (see Figure 3).

Considering the TASIT, AIHQ, and AIHQ_AMB scores, the null models were the best-fitting ones, not including fixed factors when measuring pre-post or follow-up measurements. The Supplementary materials (Section B) report the graphical presentation of non-significant effects.

#### Correlation analysis

Correlations at baseline highlighted interesting results among clinical scales and social cognition variables. The strong correlation between age and illness duration was expected. More interestingly, the illness duration positively correlated with CGI and negatively with the functioning scale. Considering the correlations between the clinical variables, the positive symptoms measured from the PANSS were positively correlated with the general psychopathological scale, negative symptoms measured through the BNSS, and CGI. The negative symptoms measured at PANSS were positively correlated with the general psychopathological scale and CGI. General psychopathology from PANSS correlated with depressive symptoms and CGI, and negative symptoms measured at BNSS correlated with the CGI. Considering the impact of clinical symptoms on patients’, only scores at general psychopathology and depressive symptoms were negatively correlated with the reported quality of life and psychological well-being. Conversely, the functioning scores rated by caregivers were negatively correlated with all clinical scales except for depression.

**Figure 4:**
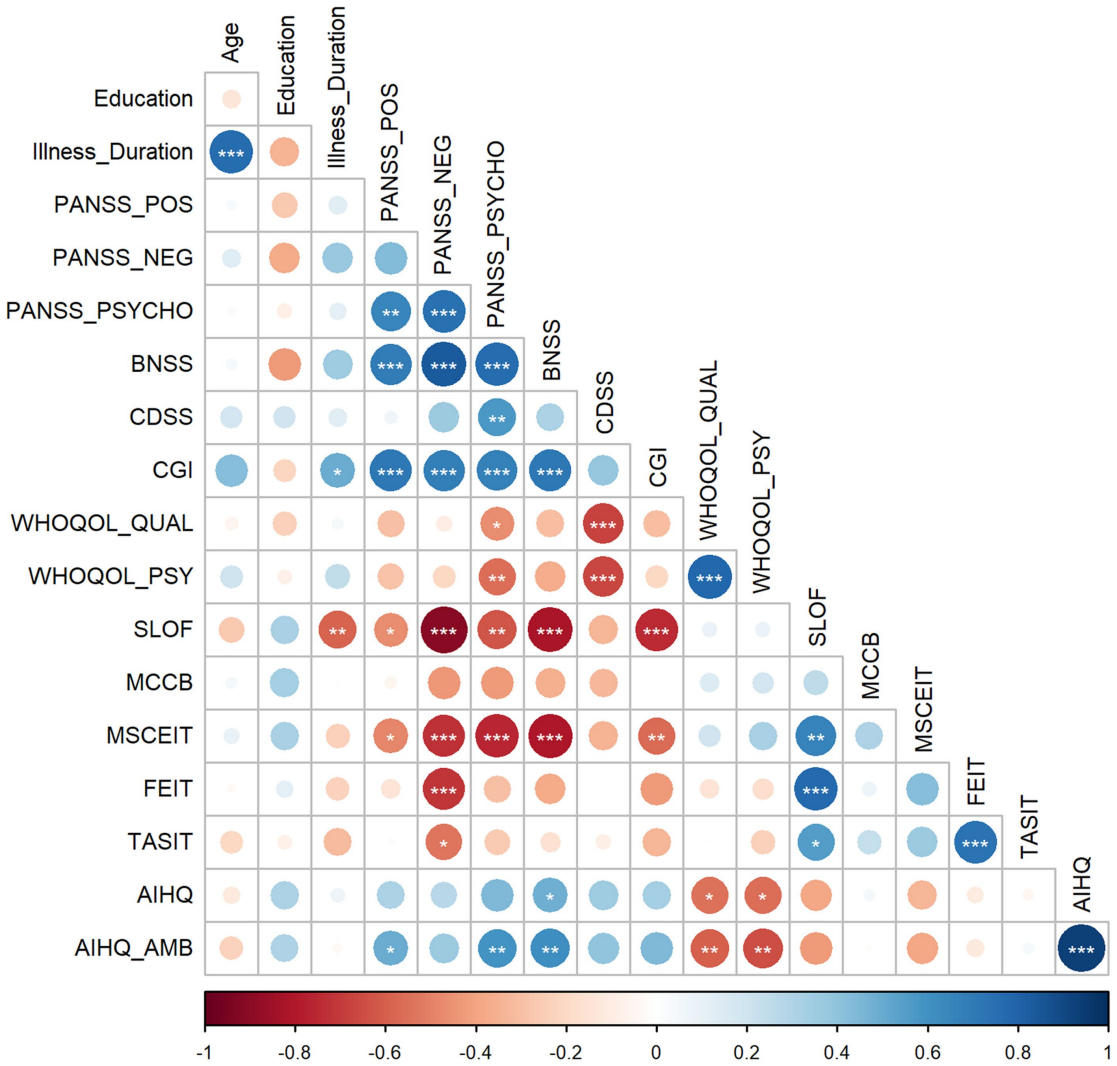
The figure shows the correlation matrix depicting the relationships between each pair of variables at the baseline. Positive correlations are blue-colored, and negative correlations are red-colored. Dots’ color intensity and size are proportional to the correlation coefficients, and asterisks inside the dots represent the statistical significance (*p < 0.05, **p < 0.01, ***p < 0.001). Note: BNSS = Brief Negative Symptoms Scale; CDSS = Calgary Depression Scale for Schizophrenia; CGI = Clinical Global Impression; PANSS_NEG = negative symptoms measured at Positive and Negative Syndrome Scale (PANSS); PANSS_POS = positive symptoms measured at PANSS; PANSS_PSYCHO = general psychopathology measured at PANSS; SLOF = Specific Level of Functioning; WHOQOL_QUAL = World Health Organization Quality of Life Assessment – quality score; WHOQOL_PSY = World Health Organization Quality of Life Assessment – psychological well-being.

As the main focus of the present work, correlations between task performance and clinical and functioning variables highlighted significant patterns. Specifically, scores at all tasks except for the attributional bias were positively correlated with the functioning scores and negatively with the negative symptoms measured through the PANSS. Moreover, emotional intelligence scores negatively correlated with negative symptoms measured through the BNSS and CGI. FEIT and TASIT were positively correlated. Scores at the ambiguous conditions in attributional bias were positively correlated with positive symptoms and general psychopathology measured through the PANSS and negative symptoms measured through the BNSS and negatively with the quality-of-life scales. Figure 4 represents the correlation matrix. Details on the statistical values of correlations (*r*) are available in the Supplementary materials (Section A).

## Discussion

In the present study, we ran a pilot randomized controlled trial to investigate the possibility of increasing the effect of a cognitive and social cognitive training through iTBS in schizophrenic patients. Participants were randomized into four groups, varying according to the stimulation condition (real vs. sham iTBS) and the behavioral intervention (no training vs. training). The protocol included 15 sessions of daily neuro-navigated iTBS, combined – or not – with 50-min individualized training, including a first 30 min that addressed the nonsocial cognitive functions (attention, verbal and visual learning, processing speed, executive functions) and 20 min of social cognition training. For the cognitive training, which is described in detail elsewhere (Vergallito et al., 2023a), we used individually targeted exercises from the Cogpack software. For the social cognitive training, we used materials from the SoCIAL in an individual setting (Palumbo et al., 2017, 2022). The social cognition training included a first module, in which we trained participants to recognize emotions from static facial pictures and short video clips. The second module included videos of everyday situations in which contextual cues (e.g., gesture and voice intonation) were crucial to understanding literal vs. nonliteral meanings of the scenes.

The clinical scales and tasks used to investigate social cognition domains were administered at five different time points, namely before and after treatment and at three follow-ups, namely one, three, and six months after the treatment end.

The novelty of our methodological approach consisted of applying a multimodal intervention, time-locking iTBS with personalized cognitive training to investigate the possibility of promoting a synergistic effect between a primer effect induced by the stimulation with the cognitive intervention. The few previous studies using NiBS to improve social cognition in schizophrenia, indeed, applied tDCS or rTMS as stand-alone interventions (Wölwer et al., 2014; Jin et al., 2023). However, it is well-established that NiBS effects are state-dependent and interact with the state of the network targeted by the stimulation, thus influencing brain activity and behavioral outcomes and possibly reducing interindividual differences (Luber and Lisanby, 2014; Romei et al., 2016; Sathappan et al., 2019; Vergallito et al., 2022; Sack et al., 2023; Vergallito et al., 2023b).

From the evidence described so far, our hypothesis was to find a general improvement in the groups receiving the training, possibly boosted in terms of effect size and duration in the group receiving the real iTBS protocol. Unfortunately, as previously reported, the whole data collection was not completed; therefore, the preliminary data presented and discussed here have a mere descriptive value and should be cautiously considered.

A first observation that deserves attention is the huge number of patients declining to participate in the study, which concerned more than 70% of the eligible patients. The main reasons for declining the treatment were related to the lack of illness insight, so that no further treatment was considered necessary, or to the feeling that protocol was too demanding. The foresight to offer transport facilities or benefits for participation in the study failed to incentivize enrollment. Interestingly, only one participant dropped out of the study after the first appointment, while the others completed the three-week protocol. The difficulty of engaging schizophrenic patients in trials is well-known (Beebe et al., 2005); indeed, it has been reported that schizophrenic patients are more likely to refuse to participate in research projects compared to patients with another psychiatric diagnosis (Carr and Whittenbaugh, 1968; Schubert et al., 1984), possibly due to the presence of negative symptoms, among which avolition, that is considered the most critical target of the treatment (Strauss et al., 2021).

Considering the primary endpoint of our study, namely changes induced by the intervention immediately after the treatment, we found a trend toward improvement in the MSCEIT-ME, which was specific for participants receiving the training. Emotional intelligence, here referred to the competence in managing emotions, was the only ability improved by the training since no differences were found in pre and post-treatment scores for facial expression recognition, ToM, and attribution of intentions. This pattern of results was unexpected since our training mostly focused on emotion recognition and ToM, whereas MSCEIT-ME measures patients’ cognitive abilities related to reasoning and problem-solving in regulating their own and other emotions (Mayer et al., 1999, 2008). Previous studies suggested that emotional intelligence and ToM abilities are conceptually linked but distinct domains (Blair, 2002). ToM requires inferring other people’s mental states in others – and not only the emotional state; conversely, emotional intelligence includes self-esteem, motivation, and regulation of own emotions, aspects which are unrelated to ToM (Ferguson and Austin, 2010). Results slightly changed when the follow-up measurements were added to the analyses, evidencing that the trend reported in emotional intelligence scores disappeared.

The lack of effect of social cognition training is not consistent with previous meta-analyses evaluating the effectiveness of similar interventions, which typically suggested an improvement, at least on emotion recognition and ToM (Grant et al., 2017; Kimoto et al., 2019; Nijman et al., 2020). However, most of the reviewed studies included one or two weekly sessions, with a total duration of about 20 sessions. Unlike these studies, our protocol was “intensive” and took place every day for three weeks. Different results in our protocol may be due to the closer sessions that prevented participants from retaining the acquired knowledge and putting it into practice. It is worth noting that the SoCIAL intervention also provided preliminary but effective results in improving social cognition abilities (Palumbo et al., 2017). However, our training had at least two main differences with the SoCIAL protocol. The first concerns the already mentioned timing and duration of the intervention. The second – and possibly even more crucial – difference is that we did not apply the module for narrative enhancement included in the SoCIAL training, which aims at improving patients’ metacognition, i.e., the ability to understand and reason on own mental states, emotions, and thought processes. Applying the narrative enhancement module, in addition to emotion recognition-ToM sections and the cognitive training, would have requested longer training duration, unfitting with the time-locked brain stimulation (Wischnewski and Schutter, 2015) and patients’ attentive resources. However, we acknowledge the importance of boosting metacognition to generalize the treatment benefits to patients’ everyday lives, and future research should carefully consider implementing its training.

Considering brain stimulation, we did not find an effect of the iTBS intervention on individuals’ performance as a standalone treatment nor combined with the training. It is possible that the activation induced by our iTBS protocol did not totally fit with the neural underpinnings involved in the trained abilities. Previous studies that applied stimulation over the left DLPFC reported improved emotion recognition in schizophrenia and major depressive disorder (Wölwer et al., 2014; Rassovsky et al., 2015; Brennan et al., 2017). Moreover, a previous sham-controlled study on healthy participants (Tik et al., 2017) suggested that high-frequency rTMS delivered over the left DLPFC increased connectivity in a frontoparietal network, including the DLPFC, the inferior frontal and dorsomedial prefrontal cortices, the dorsal cingulate cortex, the inferior parietal lobule, and the posterior temporal lobes. The frontoparietal network has been suggested to be impaired in schizophrenic patients compared to healthy controls (Deserno et al., 2012; Fryer et al., 2015; Ćurčić-Blake et al., 2022) and is involved in social cognition abilities (Green et al., 2015a; Arioli et al., 2018; Kimoto et al., 2019). Therefore, by stimulating the left DLPFC, we expected to improve its activity and connectivity with a more extended network of regions, and, in turn, modulating performance in social cognitive tasks.

We did not find effects or even trends pointing in this direction. It is possible that a different network is more directly involved in social cognition. For example, individuals judging other’s emotions (vs. a control condition such as physical judgements) recruited an extended network including the MPFC, TPJ, superior temporal sulcus (STS), precuneus, and temporal poles (Gallagher et al., 2000; Saxe et al., 2004; Dodell-Feder et al., 2011; Schneider et al., 2014; Naughtin et al., 2017). The right TPJ, in particular, seems to be a core hub of this network, involved across several ToM tasks (Saxe, 2009; Saxe and Kanwisher, 2013; Schurz et al., 2014; Boccadoro et al., 2019; Filmer et al., 2019). Furthermore, we do not exclude that more intensive protocols (for example accelerated paradigms) or a larger number of sessions are necessary to observe changes at the brain and, in turn, at the behavioral level.

The correlation analyses highlighted some interesting patterns. Besides correlations between the clinical scales, all the social cognition tasks were positively correlated with the functioning scores, namely, the higher the social cognition abilities, the higher the patients’ functioning as rated by the caregivers. Conversely, the nonsocial cognitive scores measured through the MCCB did not correlate with any of the clinical variables or performance at social cognitive tasks. These findings are in line with the large body of evidence suggesting that social cognition abilities have a closer link with real-life patients functioning than nonsocial abilities (Brekke et al., 2005; Schmidt et al., 2011; Green et al., 2015b; Halverson et al., 2019; Mucci et al., 2021). Scores at the ambiguous items in the attributional bias task were positively correlated with positive and general psychopathology scores at PANSS and negative symptoms at BNSS, suggesting that more hostile bias attribution was present in participants with higher clinical symptoms. Moreover, higher scores in attributional bias were negatively correlated with the reported psychological well-being and quality of life. Negative symptoms, instead, were negatively correlated with performance in all tasks except for attributional bias. Therefore, patients with higher negative symptoms had lower performance in social cognition tasks. Considering previous literature, correlations between positive symptoms and social cognition provided inconsistent results (Peyroux et al., 2019), with some studies reporting impairment in emotion recognition and theory of mind in patients with higher positive symptoms (Hall et al., 2004; Bora et al., 2009; Kohler et al., 2010; Montag et al., 2011), others reporting positive correlation with attributional biases, such as attributing hostile intentions to others (Green and Leitman, 2008). Research seems more consistent concerning negative symptoms, which are typically associated with greater impairment in social cognition (Brüne, 2005; Sprong et al., 2007; Lincoln et al., 2011). Considering the correlations between the administered social cognition tasks, emotion recognition and ToM abilities were positively correlated, while emotional intelligence and attributional bias did not correlate with performance at the other social cognitive tasks.

### Limitations of the present study

A clear limitation of the present study is the restricted number of participants included in the analyses. Unfortunately, the COVID-19 pandemic and the high rate of patients declining to participate in the project prevented us from completing the foreseen sample by respecting the funding time constraints.

Therefore, we acknowledge that the results presented here have a mere descriptive value, and it is impossible to disentangle whether the lack of significant results is due to the non-effectiveness of the proposed methodology or the limited number of participants completing the experiment.

## Conclusion

We presented preliminary data from a treatment combining iTBS with cognitive training. The study is feasible considering the possibility of combining brain stimulation with a cognitive intervention. Participants well tolerated the iTBS procedure without major effects such as seizures and syncopes. The most serious threat to feasibility is involving patients in the study, probably due to illness features and to our methodological procedure, which was perceived as highly demanding by participants. To overcome these issues, future studies should consider multi-centered trials and evaluate the possibility of using other NiBS techniques, such as tDCS, which can be easily transported into psychiatric facilities, perhaps reducing the experimental demand.

## Data availability statement

The datasets and the script generated for the analyses are publicly available at https://osf.io/d5aeb/. The raw data supporting the conclusions of this article will be made available by the authors, without undue reservation.

## Ethics statement

The studies involving humans were approved by Comitato Etico Milano Area 1 ASST Fatebenefratelli Sacco. The studies were conducted in accordance with the local legislation and institutional requirements. The participants provided their written informed consent to participate in this study.

## Author contributions

AV: Formal analysis, Investigation, Methodology, Writing – original draft. BG: Investigation, Writing – review & editing. KM: Writing – original draft. LG: Methodology, Resources, Writing – review & editing. DP: Methodology, Resources, Writing – review & editing. CG: Methodology, Project administration, Writing – review & editing. ST: Conceptualization, Data curation, Funding acquisition, Investigation, Methodology, Project administration, Writing – original draft, Writing – review & editing.
