# Supplementary material for: Combining transcranial magnetic stimulation with training to improve social cognition impairment in schizophrenia: a pilot randomized controlled trial
Source: Front Psychol. 2024 Feb 20;15:1308971. doi: 10.3389/fpsyg.2024.1308971 (PMC10912559; doi:10.3389/fpsyg.2024.1308971)
Supplement: Supplementary file 1 [file Table_1.pdf]

Combining transcranial magnetic stimulation with training to improve social cognition impairment in schizophrenia: a pilot randomized controlled trial.

Vergallito A., Gramano B., La Monica K., Giuliani L., Palumbo D., Gesi C. Torriero S.

Supplementary materials

Section A – Model selection

Table S1. Results of the mixed-effect analysis on MSCEIT comparing pre- vs. post-treatment scores.

Parameter	χ^2	***P***	***Removal order***	χ^2	***Df***	***p***
Time	-	-	*Not removed*	3.5526	1	.0595
Training	-	-	*Not removed*	0.6645	1	.4150
*Training*Time*	-	-	*Not removed*	3.2296	1	.0723
*Training*Stimulation*Time*	0.1877	.6649	1	-	-	-
*Stimulation*Time*	0.0044	.9471	2	-	-	-
*Stimulation*Training*	0.1320	.7164	3	-	-	-
Stimulation	0.2080	.6484	4	-	-	-

Table S1 summarizes the model-simplification procedure, including the goodness-of-fit tests and their results. The rightmost part of each table reports the effects of the included variables.

Table S2. Results of the mixed-effect analysis on MSCEIT comparing the five data points

Parameter	χ^2	***P***	***Removal order***	χ^2	***Df***	***p***
*Training*Stimulation*Time*	0.4536	.9779	1	-	-	-
*Stimulation*Time*	0.3055	.9895	2	-	-	-
*Stimulation*Training*	0.0598	.8068	3	-	-	-
Stimulation	0.2579	.6115	4	-	-	-
*Training * Time*	7.1098	.1302	5			
Training	0.5195	.4711	6			
Time	5.5540	.2350	7			

Table S2 summarizes the model-simplification procedure, including the goodness-of-fit tests and their results. The rightmost part of each table reports the effects of the included variables.

Table S3. Results of the mixed-effect analysis on FEIT comparing pre- vs. post-treatment scores.

Parameter	χ^2	P	Removal order	χ^2	Df	p
Stimulation	-	-	Not removed	0.1638	1	.6857
Training	-	-	Not removed	0.1430	1	.7053
Time	-	-	Not removed	0.9719	1	.3242
Stimulation*Training	-	-	Not removed	0.0198	1	.8882
Stimulation*Time	-	-	Not removed	1.7065	1	.1914
Training*Time	-	-	Not removed	1.5172	1	.2180
Stimulation*Training*Time	-	-	Not removed	3.8331	1	.0503

Table S3 summarizes the model-simplification procedure, including the goodness-of-fit tests and their results. The rightmost part of each table reports the effects of the included variables.

Table S4. Results of the mixed-effect analysis on FEIT, including follow-up evaluations

Parameter	χ^2	P	Removal order	χ^2	Df	p
Time	-	-	*Not removed*	8.4345	4	.0769
*Training*Stimulation*Time*	6.3647	.1735	1	-	-	-
*Stimulation*Training*	0.0340	.8537	2	-	-	-
*Stimulation*Time*	2.3389	.6737	3	-	-	-
Stimulation	0.4554	.4998	4	-	-	-
*Training*Time*	7.1885	.1263	5	-	-	-
Training	0.0064	.9362	6	-	-	-

Table S4 summarizes the model-simplification procedure, including the goodness-of-fit tests and their results. The rightmost part of each table reports the effects of the included variables.

Table S5. Results of the mixed-effect analysis on TASIT total scores before and after the treatment

Parameter	χ^2	***P***	***Removal order***	χ^2	***Df***	***p***
*Training*Stimulation*Time*	0.0696	.7919	1	-	-	-
*Stimulation*Time*	0.0206	.8859	2	-	-	-
*Stimulation*Training*	0.1045	.7464	3	-	-	-
*Training*Time*	0.2646	.6070	4	-	-	-
Stimulation	1.7989	.1798	5	-	-	-
Time	2.0812	.1491	6	-	-	-
Training	2.5887	.1076	7	-	-	-

Table S5 summarizes the model-simplification procedure, including the goodness-of-fit tests and their results. The rightmost part of each table reports the effects of the included variables.

Table S6. Results of the mixed-effect analysis on TASIT total scores, including follow-up measures

Parameter	χ^2	***P***	***Removal order***	χ^2	***Df***	***p***
*Training*Stimulation*Time*	2.5930	.6281	1	-	-	-
*Stimulation*Time*	1.0414	.9034	2	-	-	-
*Stimulation*Training*	0.1374	.7109	3	-	-	-
*Training*Time*	3.1556	.5321	4	-	-	-
Stimulation	1.8073	.1788	5	-	-	-
Training	1.223	.2689	6	-	-	-
Time	7.1143	.1300	7	-	-	-

Table S6 summarizes the model-simplification procedure, including the goodness-of-fit tests and their results. The rightmost part of each table reports the effects of the included variables.

Table S7. Results of the mixed-effect analysis on AIHQ scores comparing pre- vs. post-treatment scores.

Parameter	χ^2	***P***	***Removal order***	χ^2	***Df***	***p***
*Training*Stimulation*Time*	0.0135	.9077	1	-	-	-
*Stimulation*Training*	0.5985	.4392	2	-	-	-
*Training*Time*	1.1797	.2774	3	-	-	-
Training	1.2906	.2559	4	-	-	-
*Stimulation*Time*	1.4405	.2301	5	-	-	-
Time	0.0460	.8302	6	-	-	-
Stimulation	1.1995	.2734	7	-	-	-

Table S7 summarizes the model-simplification procedure, including the goodness-of-fit tests and their results. The rightmost part of each table reports the effects of the included variables.

Table S8. Results of the mixed-effect analysis on AIHQ scores comparing including follow-ups.

Parameter	χ^2	***P***	***Removal order***	χ^2	***Df***	***p***
*Training*Stimulation*Time*	1.6211	.8050	1	-	-	-
*Stimulation*Training*	0.2520	.6157	2	-	-	-
*Training*Time*	2.3114	.6787	3	-	-	-
*Stimulation*Time*	4.0918	.3937	4	-	-	-
Stimulation	0.2795	.5970	5	-	-	-
Time	2.0717	.7226	6	-	-	-
Training	1.1562	.2823	7	-	-	-

Table S8 summarizes the model-simplification procedure, including the goodness-of-fit tests and their results. The rightmost part of each table reports the effects of the included variables.

Table S9. Results of the mixed-effect analysis on AIHQ_AMB scores comparing pre- vs. post-treatment scores.

Parameter	χ^2	***P***	***Removal order***	χ^2	***Df***	***p***
*Training*Stimulation*Time*	0.4788	.4890	1	-	-	-
*Training*Time*	0.2729	.6014	2	-	-	-
Stimulation Time*	0.6371	.4248	3	-	-	-
*Stimulation*Training*	0.7360	.3909	4	-	-	-
Training	0.1986	.6559	5	-	-	-
Time	0.6611	.4162	6	-	-	-
Stimulation	1.9348	.1642	7	-	-	-

Table S9 summarizes the model-simplification procedure, including the goodness-of-fit tests and their results. The rightmost part of each table reports the effects of the included variables.

Table S10. Results of the mixed-effect analysis on AIHQ_AMB scores comparing including follow-ups.

Parameter	χ^2	***P***	***Removal order***	χ^2	***Df***	***p***
*Training*Stimulation*Time*	1.8840	.7644	1	-	-	-
*Training*Time*	1.5393	.8196	2	-	-	-
Stimulation Time*	3.0799	.5445	3	-	-	-
*Stimulation*Training*	0.9220	.3369	4	-	-	-
Training	0.0288	.8652	5	-	-	-
Time	4.2276	.3761	6	-	-	-
Stimulation	1.1826	.2768	7	-	-	-

Table S10 summarizes the model-simplification procedure, including the goodness-of-fit tests and their results. The rightmost part of each table reports the effects of the included variables.

Table S9. Results of the correlation analyses at baseline between clinical scale scores and social cognition tasks.

	Age	Education	Illness_Duration	PANSS_POS	PANSS_NEG	PANSS_PSYCHO	BNSS	CDSS	CGI	WHOQOL_QUAL	WHOQOL_PSY	SLOF	MCCB	MSCEIT	FEIT	TASIT	AIHQ	AIHQ_AMB
**Age**	1.00	-0.13	0.77	0.04	0.14	0.03	0.05	0.19	0.43	-0.06	0.20	-0.26	0.05	0.10	-0.02	-0.21	-0.11	-0.22
**Education**	-0.13	1.00	-0.35	-0.27	-0.37	-0.09	-0.43	0.20	-0.21	-0.23	-0.08	0.33	0.34	0.33	0.12	-0.08	0.32	0.31
**Illness_Duration**	0.77	-0.35	1.00	0.13	0.37	0.11	0.36	0.13	0.50	0.05	0.25	-0.58	0.01	-0.23	-0.23	-0.31	0.08	-0.03
**PANSS_POS**	0.04	-0.27	0.13	1.00	0.43	0.66	0.69	0.06	0.71	-0.29	-0.28	-0.47	-0.05	-0.49	-0.15	0.02	0.31	0.49
**PANSS_NEG**	0.14	-0.37	0.37	0.43	1.00	0.75	0.83	0.37	0.69	-0.09	-0.21	-0.91	-0.43	-0.72	-0.71	-0.53	0.27	0.37
**PANSS_PSYCHO**	0.03	-0.09	0.11	0.66	0.75	1.00	0.77	0.58	0.67	-0.47	-0.55	-0.63	-0.43	-0.76	-0.30	-0.26	0.44	0.59
**BNSS**	0.05	-0.43	0.36	0.69	0.83	0.77	1.00	0.32	0.71	-0.30	-0.37	-0.81	-0.35	-0.80	-0.38	-0.16	0.48	0.61
**CDSS**	0.19	0.20	0.13	0.06	0.37	0.58	0.32	1.00	0.38	-0.68	-0.67	-0.34	-0.32	-0.34	0.00	-0.08	0.35	0.40
**CGI**	0.43	-0.21	0.50	0.71	0.69	0.67	0.71	0.38	1.00	-0.30	-0.21	-0.75	0.00	-0.57	-0.43	-0.33	0.33	0.44
**WHOQOL_QUAL**	-0.06	-0.23	0.05	-0.29	-0.09	-0.47	-0.30	-0.68	-0.30	1.00	0.79	0.08	0.15	0.19	-0.15	0.00	-0.55	-0.60
**WHOQOL_PSY**	0.20	-0.08	0.25	-0.28	-0.21	-0.55	-0.37	-0.67	-0.21	0.79	1.00	0.08	0.19	0.32	-0.18	-0.24	-0.55	-0.64
**SLOF**	-0.26	0.33	-0.58	-0.47	-0.91	-0.63	-0.81	-0.34	-0.75	0.08	0.08	1.00	0.25	0.66	0.77	0.55	-0.39	-0.43
**MCCB**	0.05	0.34	0.01	-0.05	-0.43	-0.43	-0.35	-0.32	0.00	0.15	0.19	0.25	1.00	0.30	0.07	0.24	0.05	-0.01
**MSCEIT**	0.10	0.33	-0.23	-0.49	-0.72	-0.76	-0.80	-0.34	-0.57	0.19	0.32	0.66	0.30	1.00	0.43	0.37	-0.34	-0.39
**FEIT**	-0.02	0.12	-0.23	-0.15	-0.71	-0.30	-0.38	0.00	-0.43	-0.15	-0.18	0.77	0.07	0.43	1.00	0.74	-0.11	-0.12
**TASIT**	-0.21	-0.08	-0.31	0.02	-0.53	-0.26	-0.16	-0.08	-0.33	0.00	-0.24	0.55	0.24	0.37	0.74	1.00	-0.04	0.05
**AIHQ**	-0.11	0.32	0.08	0.31	0.27	0.44	0.48	0.35	0.33	-0.55	-0.55	-0.39	0.05	-0.34	-0.11	-0.04	1.00	0.93
**AIHQ_AMB**	-0.22	0.31	-0.03	0.49	0.37	0.59	0.61	0.40	0.44	-0.60	-0.64	-0.43	-0.01	-0.39	-0.12	0.05	0.93	1.00

Table S9 summarizes the Pearson correlation coefficients (r) for each couple of variables. P-values are reported in the caption of Figure 4.

Note: BNSS = Brief Negative Symptoms Scale; CDSS = Calgary Depression Scale for Schizophrenia; CGI = Clinical Global Impression; PANSS_NEG = negative symptoms measured at Positive and Negative Syndrome Scale (PANSS); PANSS_POS = positive symptoms measured at PANSS; PANSS_PSYCHO = general psychopathology measured at PANSS; SLOF = Specific Level of Functioning; WHOQOL_QUAL = World Health Organization Quality of Life Assessment – quality score; WHOQOL_PSY = World Health Organization Quality of Life Assessment – psychological well-being.

Section B – Supplementary graphical presentation

Figure S1. Individual trends in emotion recognition scores (FEIT task) at different time points.

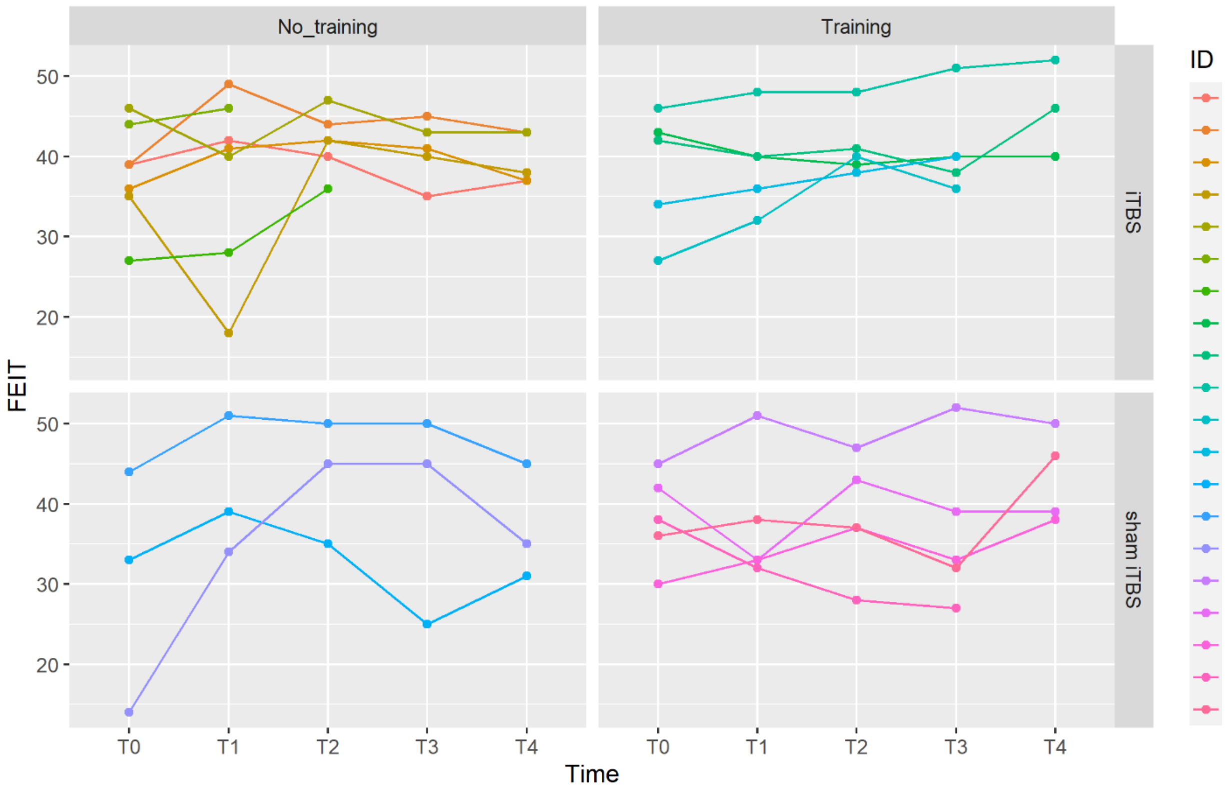

Figure S1. The figure depicts individuals' performance in the emotion recognition task at different time points. Subjects are grouped by stimulation and training conditions. In the group receiving iTBS + training, one participant performed only the MSCEIT-ME task instead of the complete social cognition assessment due to his high fatigability. Therefore, only five participants are displayed in the figure.

Figure 2. Theory of mind scores trend.

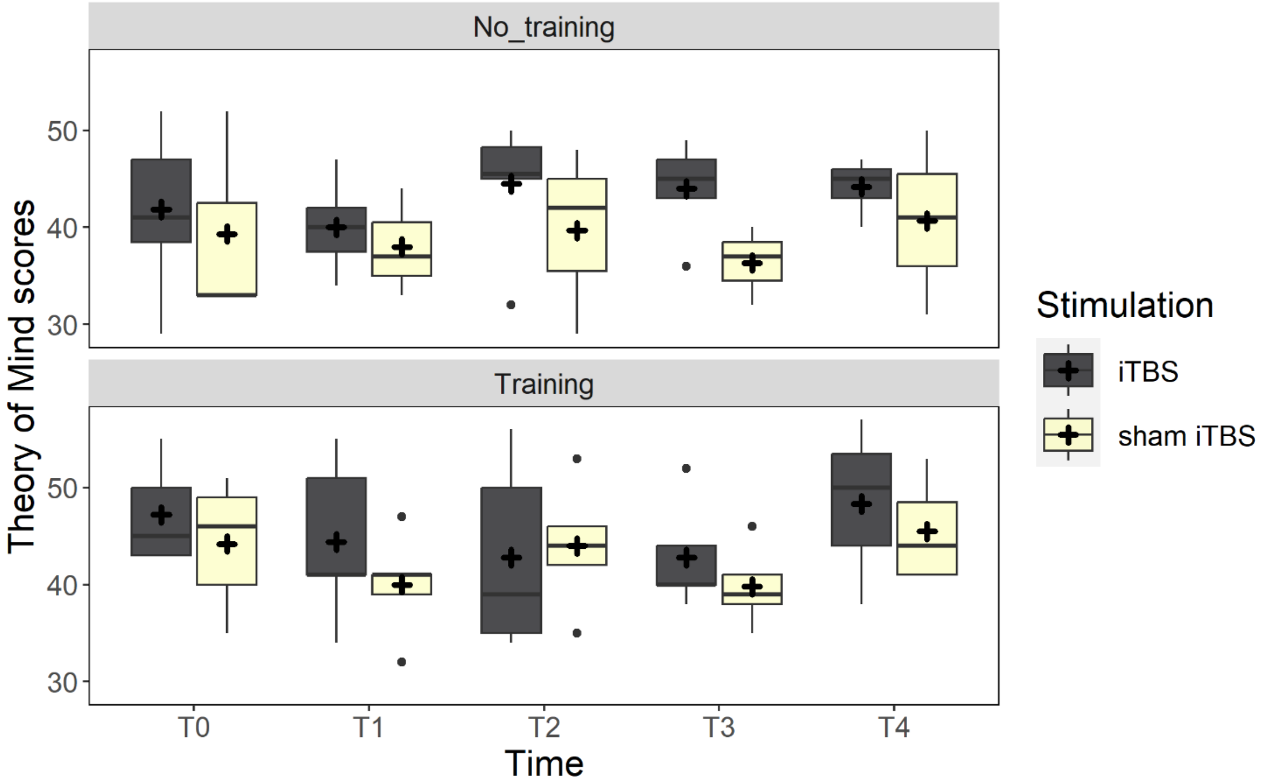

Figure S2. The figure depicts the theory of mind scores (TASIT task) at different time points. The boxplots separately compare real iTBS (dark gray boxes) and sham iTBS (light yellow boxes) for the no training and training conditions.

Figure S3 – Attributional bias scores trend.

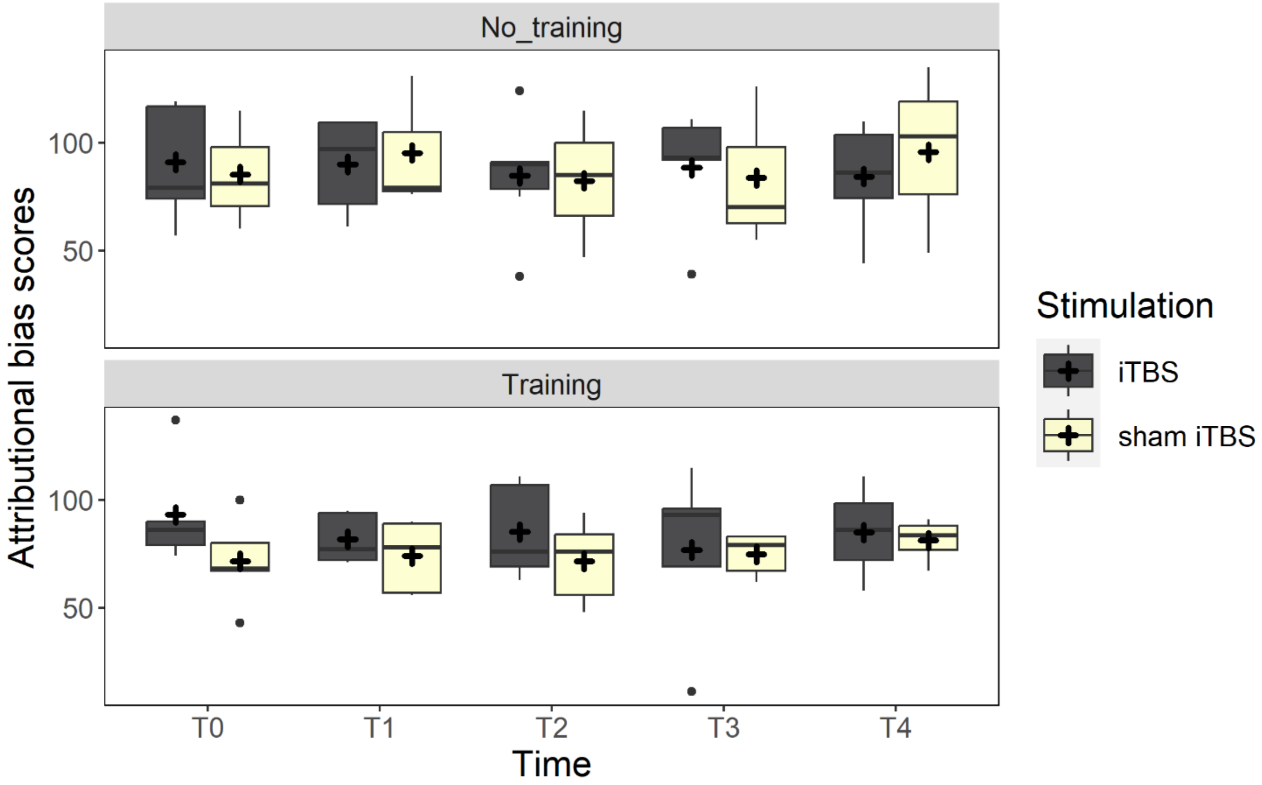

Figure S3. The figure depicts the attributional bias scores (AIHQ task) at different time points. The boxplots separately compare real iTBS (dark gray boxes) and sham iTBS (light yellow boxes) for the no training and training conditions.

Figure S4 – Attributional bias –ambiguous items' scores.

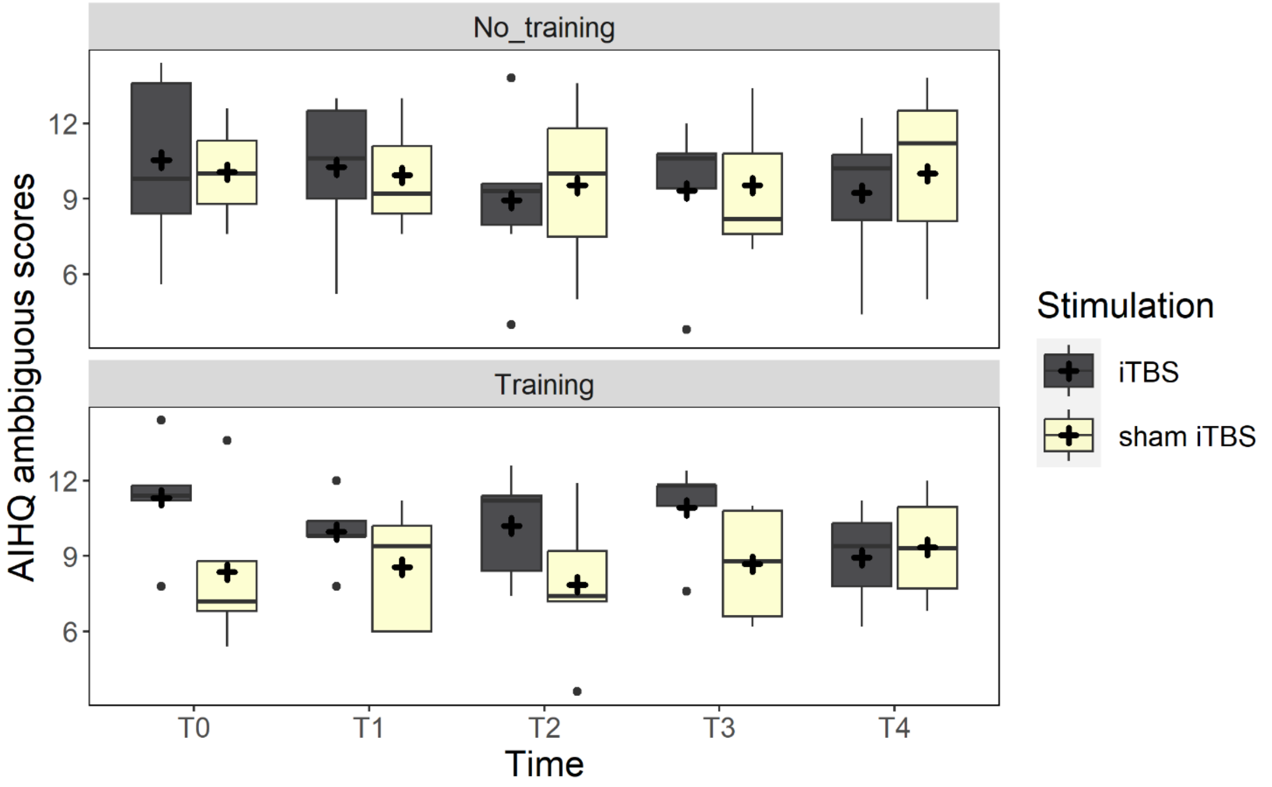

Figure S4. The figure depicts the scores for the ambiguous items at the attributional bias task (AIHQ) at different time points. The boxplots separately compare real iTBS (dark gray boxes) and sham iTBS (light yellow boxes) for the no training and training conditions.
